# Scaffold proteins in cancer

**DOI:** 10.18632/oncoscience.177

**Published:** 2015-07-28

**Authors:** Jie Xu, Jing-Yuan Fang

**Affiliations:** Key Laboratory of Gastroenterology & Hepatology, Ministry of Health; Division of Gastroenterology and Hepatology, Renji Hospital, School of Medicine, Shanghai Jiao Tong University, China

**Keywords:** scaffold protein, protein modification, proliferation, gastrointestinal cancers

Scaffold proteins are crucial regulators of many key signaling pathways that function by interacting with multiple members of a signaling pathway and tethering them into complexes. We recently identified novel scaffold proteins that respectively regulate p53 and ERK pathways in gastrointestinal tumors. ArhGAP30 is a member of the RhoGAP family that are known to regulate Rho GTPases and thereby affect actin remodeling and cell motility. However, our recent study reported a RhoGAP-independent function of ArhGAP30 [1], which is implicated in regulating p53 posttranslational modification (PTM). ArhGAP30 binds both p300 and p53, facilitating p300-mediated acetylation of p53 C-terminal residue K382. This substantially increases p53 transcriptional function and promotes cell cycle arrest and apoptosis.

Likewise, we also found a non-canonical function of synbindin, which is a subunit of TRAPP (TRAnsport Protein Particle) complex involved in particle transport between organelles [2]. We found that synbindin functions as a scaffold protein that interacts with MEK and ERK on the Golgi apparatus. The interaction promotes phosphorylation of ERK by MEK, and induces cell proliferation and migration. The synbindin LDc domain was found to bind ERK DEF domain, as revealed by our following study [3]. Although the biological roles of ArhGAP30 and synbindin are distinct (anti- and pro-proliferation), their molecular functions are both scaffold proteins that regulate protein PTM.

**Figure 1:**
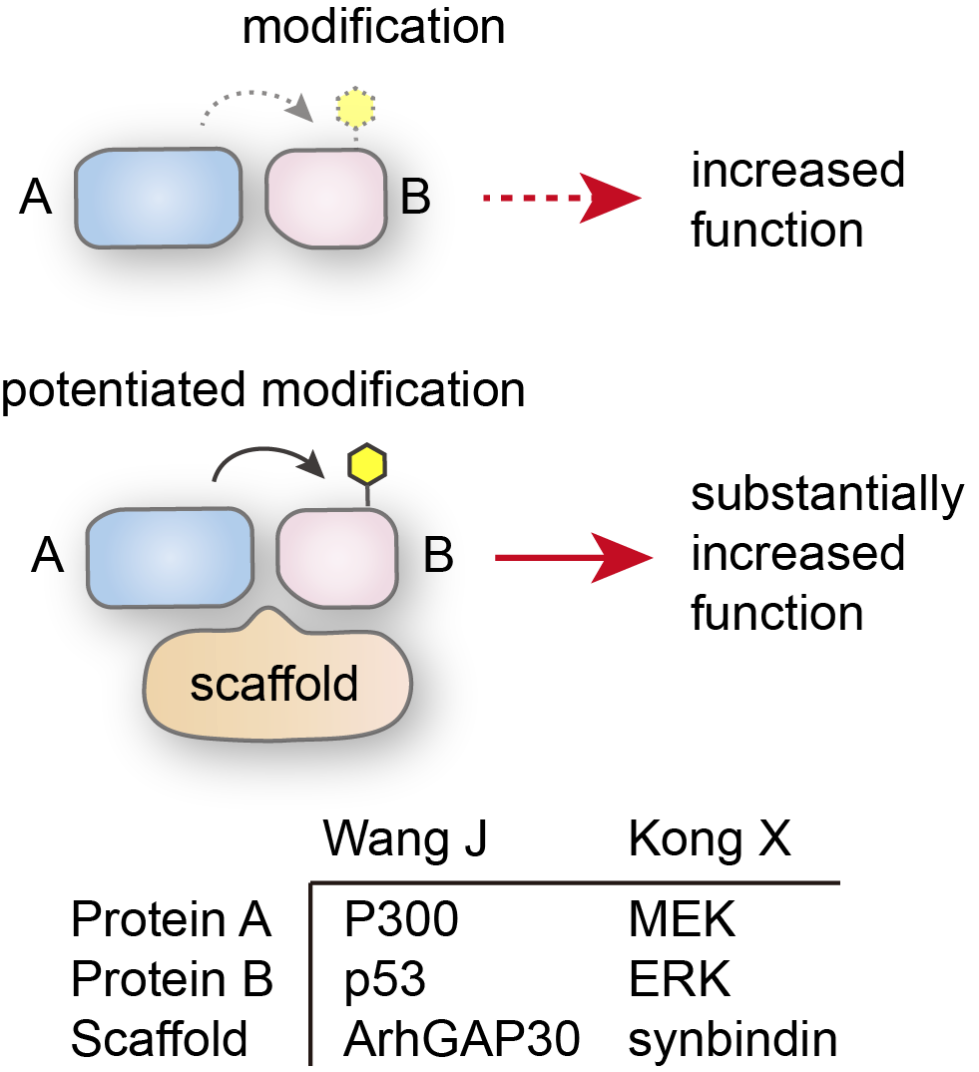
Schaffold protein stimulates the modification of protein B by protein A The examples for both proteins are shown in the lower panel.

Interestingly, these PTM-related roles do not seem to require their canonical functions, i.e., the RhoGAP enzyme activity of ArhGAP30 and the vesicle trafficking function of synbindin. Rather, these proteins mediate the interactions of PTM kinase and client proteins by structural domains that were previously uncharacterized. Also, the functions of these scaffold proteins add to the complexity of PTM processes, as both p53 acetylation and ERK phosphorylation have been reported to be under regulation of various factors [4, 5]. It remains to be clarified which of these reported mechanisms may represent the predominant route for p53 and ERK PTMs in a specific tissue type or disease status.

The scaffold protein-mediated PTM may help to explain the prevalence of deregulated p53 and ERK PTM in cancers. As an example, ERK phosphorylation has been found in most gastric cancer tissues, but the alterations in canonical components (such as Ras, Raf and Mek) can only explain some of the abnormal ERK phosphorylation [6]. The finding that synbindin may contribute to ERK phosphorylation shed light on the multifaceted mechanisms underlying ERK hyperactivation, and indicates alternative therapeutic opportunities by targeting scaffold proteins.
